# Molecular identification and phylogenetic analysis of a *Callosciurus notatus* complete mitogenome from Peninsular Malaysia

**DOI:** 10.1080/23802359.2020.1797583

**Published:** 2020-08-26

**Authors:** Puteri Nur Syahzanani Jahari, Shahfiz Mohd Azman, Kaviarasu Munian, Nor Hazwani Ahmad Ruzman, Mohd Shahir Shamsir, Stine R. Richter, M. Thomas P. Gilbert, Faezah Mohd Salleh

**Affiliations:** a Department of Biosciences, Faculty of Science, Universiti Teknologi Malaysia, Johor Bahru, Malaysia; b Forest Biodiversity Division, Forest Research Institute Malaysia, Kepong, Malaysia; c Faculty of Applied Sciences and Technology, Universiti Tun Hussein Onn Malaysia, Pagoh Higher Education Hub, Muar, Malaysia; d Section for Evolutionary Genomics, The GLOBE Institute, University of Copenhagen, Copenhagen, Denmark

**Keywords:** *Callosciurus notatus*, mitogenome, phylogenetic analysis

## Abstract

The mitogenome of a plantain squirrel, *Callosciurus notatus*, collected from Bukit Tarek Forest Reserve (Extension), Selangor, Malaysia was sequenced using BGISEQ-500RS technology. The 16,582 bp mitogenome consists of 13 protein-coding genes, 22 transfer RNA genes, 2 ribosomal RNA genes, and 1 control region. A phylogenetic and BLASTn analysis against other available datasets showed that the mitogenome matched with 99.49% similarity to a previously published *C. notatus* mitogenome from Peninsular Malaysia. However, it also diverged by nearly 8% (92.24% match) from a second previously published mitogenome for the same species, sampled in East Kalimantan, Indonesia. This suggests a difference in landscape features between both localities might affect its genetic connectivity.

*Callosciurus notatus* is a diurnal and arboreal squirrel species (Saiful and Nordin 2004) that is abundant and widespread in Thailand, Peninsular Malaysia and across the islands of Java, Bali, Lombok, and Borneo (Bunsong Lēkhakun & McNeely, 1977 ; Thorington and Hoffmann 2005). Small mammals such as *C. notatus* are important species for the functioning of many ecosystems. They serve not only as premier food for mammalian carnivores, but are also important pollinators (Goldingay et al. 1991; Tucker and Rogers 2014) and seed dispersal (Bobadilla et al. 2016). Generation of complete mitogenome for the species could be useful resources for future investigations into their evolution and radiation across their habitat range.

The specimen sequenced here (voucher no.: MZF1962) was collected from Bukit Tarek Forest Reserve (Extension), Selangor, Malaysia (3.48 N 101.47 E) in January 2018 (Munian et al. 2020) and is currently stored at the Forest Research Institute Malaysia (FRIM). The methodologies used for DNA isolation, library construction, read assembly, and gene annotation are described in Mak et al., (2017). The mitogenome of *C. notatus* from this study (MT231329) is a circular molecule with 16,582 bp in length. Similar to the mitogenome of other *Callosciurus* species, it contained 13 protein-coding genes (PCGs), 22 transfer RNA genes, 2 ribosomal RNA genes, and 1 control region (Hu et al. 2016; Mohd Salleh et al. 2017).

The overall nucleotide composition of the *C. notatus* mitogenome reported in this study is 31.64% A, 29.76% T, 12.91% G, and 25.69% C, which showed a slight AT bias (61.40%), similar to other vertebrate mitogenomes (Mohd Salleh et al. 2017). The total length of the PCG sequences is 11,400 bp. The total length of the 22 tRNA genes is 1514 bp, ranging from 58 bp (tRNASer) to 74 bp (tRNALeu). The 12S rRNA gene length is 967 bp and the 16S rRNA gene length is 1574 bp, they are located between the tRNAPhe and tRNALeu, and are separated by the tRNAVal gene. The control region size is 71 bp and is located between tRNAPro and tRNAPhe genes. The genes are mostly located on the heavy (H) strand except NAD6 and eight tRNAs genes (tRNAGln, tRNAAla, tRNAAsn, tRNACys, tRNATyr, tRNASer, tRNAGlu, and tRNAPro), which were found to be located on the lower (L) strand.

When compared against the other mitogenomes available in GenBank, the best BLASTn hit (99.49%) was against a previously sequenced *C. notatus* (KY117541.1) mitogenome, that also originated in Peninsular Malaysia. In contrast, it differed considerably (92.24% match) to a second *C. notatus* (KY117542.1) that originated from East Kalimantan, Indonesia (Mohd Salleh et al. 2017). This is also clearly mirrored in a phylogenetic analysis showing the relationship of these three mitogenomes with those other *Callosciurus* species (Figure 1). This finding is consistent with the landscape of S.E. Asia, and in particular the marine barriers between the different land masses, which almost certainly affect the dispersal and genetic connectivity for small mammals such as *Callosciurus* species (Oshida et al. 2011; Brunke et al. 2019). In addition, *Callosciurus notatus* has also been reported as a genetically diverse species subdivided into several subspecies around Southeast Asia (Chah 2007; Sari et al. 2020). Therefore, it is clear that future comparative mitogenome studies on sample datasets spanning the full range will reveal considerable information about the origin and spread of this species across its range.

**Figure 1.**
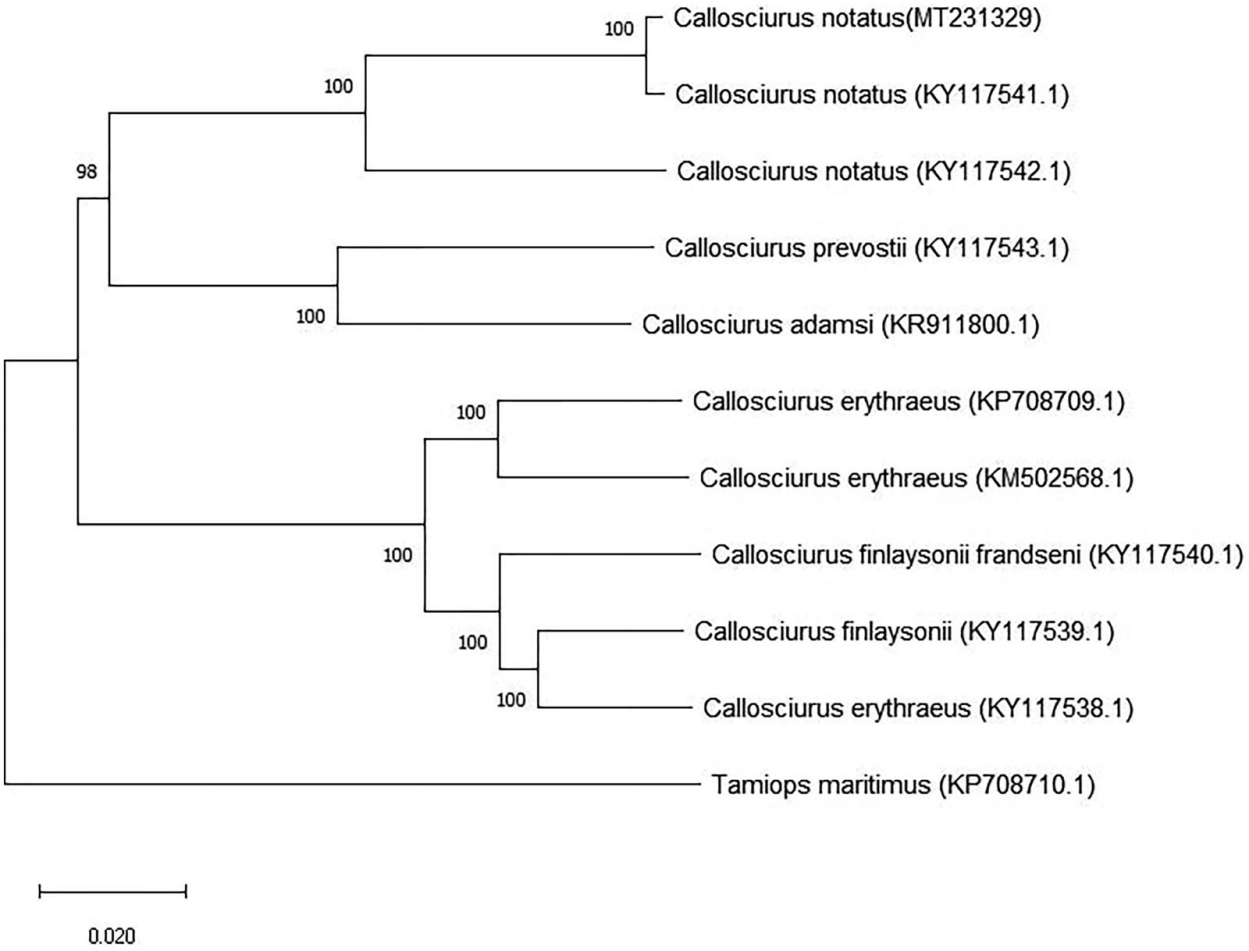
The phylogenetic tree of *C. notatus* (MT231329) mitogenome and other *Callosciurus* species available in GenBank.

## Data Availability

The data that support the findings of this study are openly available in National Center for Biotechnology Information (NCBI) at https://www.ncbi.nlm.nih.gov, accession number MT231329.
